# Breeding progress of grain and forage maize in long-term variety trials compared to on-farm yield development

**DOI:** 10.1007/s00122-025-05085-6

**Published:** 2025-11-13

**Authors:** F. Laidig, T. Feike, H. Brandes, H-P. Piepho

**Affiliations:** 1 Biostatistics Unit, Institute of Crop Science, University of Hohenheim, Fruwirthstrasse 23, 70599 Stuttgart, Germany; 2 Julius Kühn Institute – Federal Research Centre for Cultivated Plants, Institute for Strategies and Technology Assessment, Stahnsdorfer Damm 81, 14532 Kleinmachnow, Germany; 3 Bundessortenamt, Osterfelddamm 60, 30627 Hannover, Germany

## Abstract

**Key message:**

Yield gaps between variety trials and on-farm yields show diverging trends for grain versus forage maize in Germany in 1987–2023. The yield gap between variety trials and on-farm reduced for grain yield from 30% (1987) to 23% (2023), while for forage maize dry matter yield increased from 15% (1987) to 32% (2023). In variety trials during the same period starch yield in forage maize showed a moderate increase of 14%, while digestibility and starch content did not change over time. N use efficiency in variety trials was improved by 35%, 27% and 18% for grain, dry matter and starch yield, respectively, while N yield in dry matter did not change over time.

**Abstract:**

Maize cultivation increased significantly in Germany over the past 25 years. With a share of over 20% of arable land, maize has become the second most important crop after wheat, primarily due to the growing demand for biogas production. Based on long-term variety trials for grain and forage maize, we quantified breeding progress applying mixed linear models extended by linear and nonlinear regression terms to estimate time trends between 1987 and 2023. Grain yield increased by 33.4 dt ha^−1^ (36.3%) and dry matter yield of forage maize by 36.1 dt ha^−1^ (19.9%) compared to 1987. Over the last 15 years, there has been a slowdown in upward yield trends. In addition, the NUE of grain and forage maize increased by 35.0% and 27.2%, respectively. From 1987 to 2023, grain yield gaps between variety trials and national on-farm yields reduced from 30.1 to 22.6% while the stagnation of on-farm forage maize yields resulted in an increased yield gap from 15.1 to 32.1%. This diverging trend can be attributed to a complex set of reasons, such as climate change, management practices and economic constraints. Looking at quality traits in the variety trials, starch content and digestibility of forage maize did not change, but starch yield (14.0%) and NUE of starch yield (18.3%) increased, while N yield of forage maize decreased by − 4.7%, though not significant. Our study shows that breeding progress of grain maize was successfully transferred into increasing on-farm yields, while a considerable yield gap remains for forage maize, what calls for additional research.

**Supplementary Information:**

The online version contains supplementary material available at 10.1007/s00122-025-05085-6.

## Introduction

Maize (*Zea mays*), also referred to as corn, is the third most important crop grown worldwide after wheat and rice (FAOSTAT 2024). Maize is grown either for its grain, used for animal feed, industrial use or human nutrition, or for its biomass, used as silage for animal feed and for biogas production. Since the introduction of hybrid varieties in the 1930s, grain yields in the US increased from 15 dt ha^−1^ to 113 dt ha^−1^ in 2023 corresponding to an increase of 1 dt per ha and year (USDA-NASS 2024). In Germany, where maize is a popular spring crop, grain maize yielded 27 dt ha^−1^ in the 1930s (average 1935–1938), while in 2023 grain yield was nearly fourfold with 97 dt ha^−1^. Ruiz et al. (2023) reported that in the US Corn Belt, maize grain yields increased from 52 dt ha^−1^ (1970) to 111 dt ha^−1^ (2020) corresponding to an increase of 114%. This increase is the result of changes in various factors, particularly genetics, management, and the environment.

Looking at forage maize, dry matter yield was 129 dt ha^−1^ in Germany in 1955, when the national statistics started, and rose up to 147 dt ha^−1^ in 2023 (BMEL 2025). The first hybrid varieties released in Germany in 1956 were double crosses, in 1970 the first three-way cross was released and in 1973 the first single cross. Today nearly all varieties on the German market are single crosses.

**Fig. 1:**
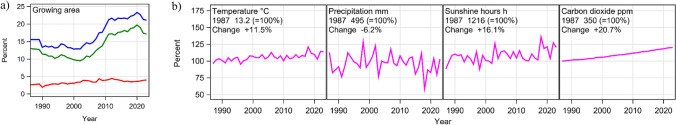
**a** Growing area of grain maize (red line), forage maize (green line) and total (blue line) as percent of total arable land in Germany. Data until 1989 are for West Germany and from 1990 on for Germany **b** Trends of average daily temperature and precipitation, cumulative annual sunshine hours, and atmospheric carbon dioxide concentration from April to October in Germany

Maize growing area in Germany is the second largest in the EU after France (Eurostat 2025). Maize accounts for more than 20% of total arable land in Germany. It is the crop with the second largest share after winter wheat with about 25% and before winter oilseed rape with less than 9%. In 1985, maize covered around 15% of arable land followed by a slight reduction until 2002. In the following years, a sharp rise in forage maize cultivation increased the share to over 20% by 2012, after which it increased only moderately (Fig. 1a). In the last years, the grain maize area stagnated below 5% (BMEL 2025; Genesis 2025). The main reason for this drastic increase in the area of ​​land used for forage maize was the sharply increasing demand for bioenergy production and the high biomass productivity of maize (Hermann et al. 2014; Rath et al. 2015). The expansion of maize cultivation, mainly in northern Germany, was made possible particularly by hybrid breeding for early maturity and cold tolerance in the juvenile stage of the plants (Laidig et al. 2014). Today the maize acreage used for bioenergy is estimated to about 45% of total maize acreage corresponding to about 0.9 million hectares (FNR 2025, Fachagentur Nachwachsende Rohstoffe e. V. (FNR)).

The reported on-farm yield gains are likely a result of both improved agronomic practices and successful plant breeding based on the introduction of new hybrid varieties allowing for higher N fertilizer and increasing plant densities (Duvick and Cassman 1999; Duvick 2005; Smith et al. 2014). In addition to higher yields, breeding has also altered other functional traits (DeBruin et al. 2017; Mueller et al. 2019; Smith et al. 2014; Taube et al. 2020), i.e., increased total biomass and N accumulation at maturity, longer stay-green leave area, improved capture of photosynthetically active radiation, decreased grain N concentration, increased plant height, and improved harvest index. Ruiz et al. (2023) reported an increase in harvest index of about 15% of the historical grain yield increase in the US Corn Belt over the last 50 years. However, prospects that the genetic yield potential could be raised further by increasing harvest index (HI) is considered small (Fischer and Edmeades 2010). Major management factors contributing to higher yields are likely increased and improved use of fertilizers, improved weed control and improved sowing techniques. Climate change may be another factor for increasing maize yields, especially increased total rainfall and increasing temperatures, especially during early growth phases (Assefa et al. 2012). As such, global warming led to an elongated vegetative period, which allowed growing varieties with higher degree-day requirements and higher yield potentials. Accordingly, Taube et al. (2020) reported that higher temperature indirectly contributed to higher yields due to the selection of maize varieties with more favorable functional traits, like higher number of leaves and longer leaves, higher radiation use efficiency and lower leaf angle.

It is important that the breeding progress observed for field crops can be translated into increasing on-farm yield, i.e., that the difference between trial and on-farm yields is small. The so-called yield gap describes the difference between a crop’s attainable yield, as, e.g., demonstrated in variety trials, and a crop’s actual yield obtained under on-farm conditions (Fischer and Edmeades 2010; Van Ittersum et al. 2013). Narrowing yield gaps especially by improved site-specific crop management and cultivar selection is considered a major driver for sustainable intensification in crop production.

Looking at its environmental impact, intensive maize cropping is associated with negative environmental effects arising from high N fertilization in conjunction with a high risk of N losses to ground water and to the atmosphere (Peichl et al. 2019; Struck et al. 2019; Bos et al. 2013). N recovery efficiency in US grain maize was reported to be below 50%, meaning less than 50% of applied N fertilizer is used by the recipient crop. This means that more than 50% of applied fertilizer remains in the soil or is lost to the environment (Cassman et al. 2002; Ciampitti et al. 2012; Lassaletta et al. 2014). Furthermore, while maize is self-compatible, the frequent cropping of maize on the same field likely leads to a reduction in soil organic matter (Komainda et al. 2018). Considering its substantial acreage, it is of high importance to reduce the negative environmental impact of maize cultivation through improved varieties and crop management to enhance the overall sustainability of crop production.

Therefore, the overall goals of this study are (i) to evaluate the breeding progress of yield and yield related traits in grain and forage maize, (ii) to quantify the gap between trial and national on-farm yields by considering trial-specific and environmental conditions, (iii) to quantify nitrogen use efficiency (NUE) and (iv) to evaluate the progress of N uptake and feeding quality of forage maize.

## Materials and methods

### Maize variety trials

This study builds on data from official maize variety trials conducted by the Federal Plant Variety Office (Bundessortenamt, Hannover) during 1987–2023 to assess the value for cultivation and use (VCU). Maize data are evaluated separately for grain and forage utilization. Maize varieties are grown under rainfed conditions in separate trial series grouped into an early, medium and late maturity group for each utilization direction. Grouping into these maturity groups is based on grain and forage maturity scores. For grain varieties, scoring depends on the dry matter content in grain at harvest and for forage varieties on the whole plant dry matter content at harvest (BSL 2024). Before 2002, all maize varieties, no matter whether they were applied for registration as grain or forage utilization varieties, had to be tested in the forage trial series. From 2003 on, testing in the forage series was no longer obligatory. From that time on, fewer grain maize varieties were included in the forage trial series, but still at least half of the varieties tested in the forage maize trial series were also tested in the grain maize trial series (Table 1). Since 1997, the regular testing period for a newly applied variety was two years while until 1996 varieties were tested for three years. Varieties in the same testing years were grown in separate trial series, i.e., in three series (S1, S2 and S3) until 1996 and later only in two series (S1 and S2) per maturity group and utilization direction. This means that combined for grain and forage maize in total 12 trial series (18 before 1997) were grown in parallel each year. Trials were conducted following “good local agronomic practice (GLAP),” implying adequate herbicide application and nutrient supply from manure and mineral fertilizers according to agronomic recommendations and within environmental application standards. Varieties in the first testing year are grown on trial sites provided and managed by seed companies. All varieties within a trial received the same treatment. N fertilizer rates include mineral and organic fertilizers. In the case of organic fertilizers, the equivalent of mineral N was either calculated based on laboratory results or approximated by standard conversion factors for different organic fertilizers. At least three reference varieties were included in each trial series and updated on a regular basis, ensuring at least partial overlap of sets of references used in successive years. To get a link between maturity groups, since 2003 at least one border variety linking to earlier or later maturity groups was included in each maturity group. Trials were laid out either as randomized complete blocks or as alpha-lattice design with three replications. The target plant density per plot was 7–11, 7–10 and 6–9 for the early, medium and late maturity group, respectively. Only the two center rows per plot of at least 7.8 m^−2^ or at least 60 plants with a row distance between 60 and 80 cm were harvested to eliminate border effects. Front and back parts of the plots were harvested. To reduce border effects at the front/back, the distance between plots had to be less than one meter. For some of the trials even a lower distance was reached or two border rows rectangular to the plots were sown and removed before harvest. Grain maize harvest was targeted when dry matter content in the grain reached at least 65%, while forage maize has an optimal harvest window with a dry matter content of the whole plant between 32 and 38% (BSA 2024).

**Table 1:** Data set

				Number of			
Trait	Maturity	First year	Years	Observations	Trials	Varieties	Common varieties
Grain maize							
Grain yield	Early	1987	37	11,218	1,063	186	132
	Medium	1987	37	14,209	1,028	274	179
	Late	1987	37	7,444	854	126	78
Harvest index	Early	1986	35	1,988	269	128	
	Medium	1986	35	3,403	354	175	
	Late	1986	35	2,628	474	77	
Forage maize							
Dry matter yield	Early	1987	37	12,244	1,021	235	132
	Medium	1987	37	16,449	1,092	307	179
	Late	1987	37	8,770	890	157	78
N yield in dry matter	Early	1999	25	6,118	501	173	
	Medium	1999	25	8,836	534	237	
	Late	1999	25	5,109	444	136	

### Traits evaluated

For grain maize, we evaluated grain yield dt ha^−1^ at 86% dry matter content of grain, harvest index, dry matter content % at harvest, thousand grain mass g at 86% dry matter content, plant height cm measured at the end of flowering, lodging plants at harvest %, days from sowing to female flowering, and NUE for grain yield in kg kg^−1^ determined as the ratio of grain yield and available N fertilizer including soil mineralized N (Nmin). For forage maize, we evaluated dry matter yield dt ha^−1^ of the whole plant without stover, dry matter content at harvest %, plant height cm, days from sowing to female flowering, lodging plants at harvest %. For forage maize, data for the following traits were available from 1999 on: crude protein content % of whole plant dry matter, N yield in dry matter derived from dry matter yield and N content % multiplied by the ratio of protein content of whole plant dry matter and protein equivalent factor of N 6.25, digestibility % of whole plant dry matter assessed by enzyme soluble organic substance (ELOS), starch content % of whole plant dry matter and starch yield dt ha^−1^. For more details see the chapter on maize in the guidelines for VCU testing of grain and forages maize (BSA 2024). In our study, the yield gap was derived as percent of the absolute difference between the VCU trial yield, considered as the attainable yield and on-farm yield from an annual harvest survey (BMEL 2025) relative to trial yield (100%). NUE was assessed for grain yield, total dry matter yield, N yield and starch yield in total plant dry matter produced per kg of available N to the crop in kg kg^−1^. For a more detailed description of NUE and its components see, e.g., Moll et al. (1982), Good et al. (2004), and Hawkesford and Ritchie (2020).

### Climatic change

Temperature, precipitation, sunshine hours and atmospheric carbon dioxide concentration in Germany during April to October, depicted in Fig. 1b (DWD 2025a; Lan et al. 2025), show increasing trends from 1987 to 2023, except for precipitation. Large year-to-year variations can be observed for precipitation and sunshine hours. An extremely dry year occurred in 2018, with high temperatures and many sunshine hours.

### Trial site conditions

**Fig. 2:**
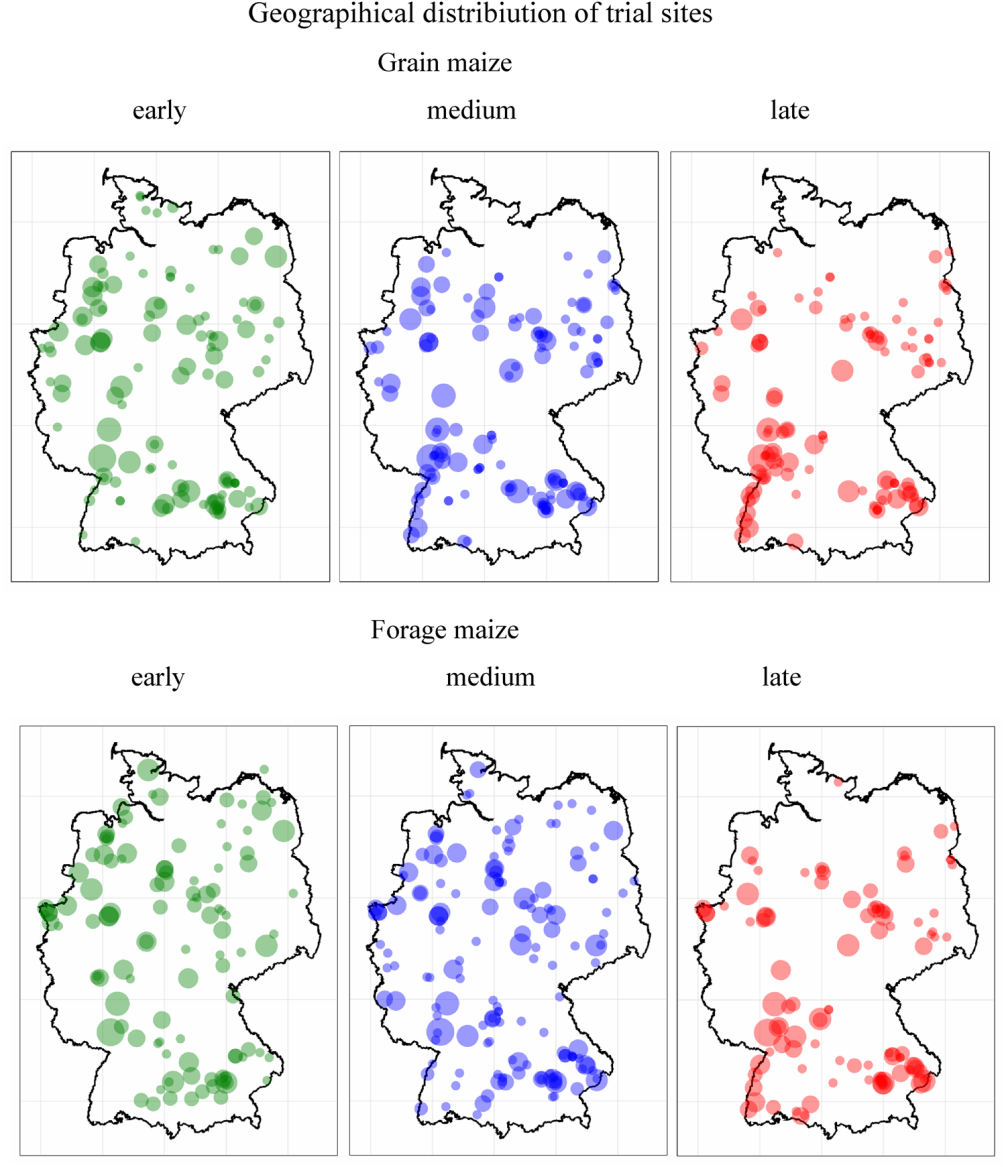
Geographical distribution of grain and forage maize trial sites. The bubble size indicates the frequency of trials grown at the same site 1987–2023

**Fig. 3:**
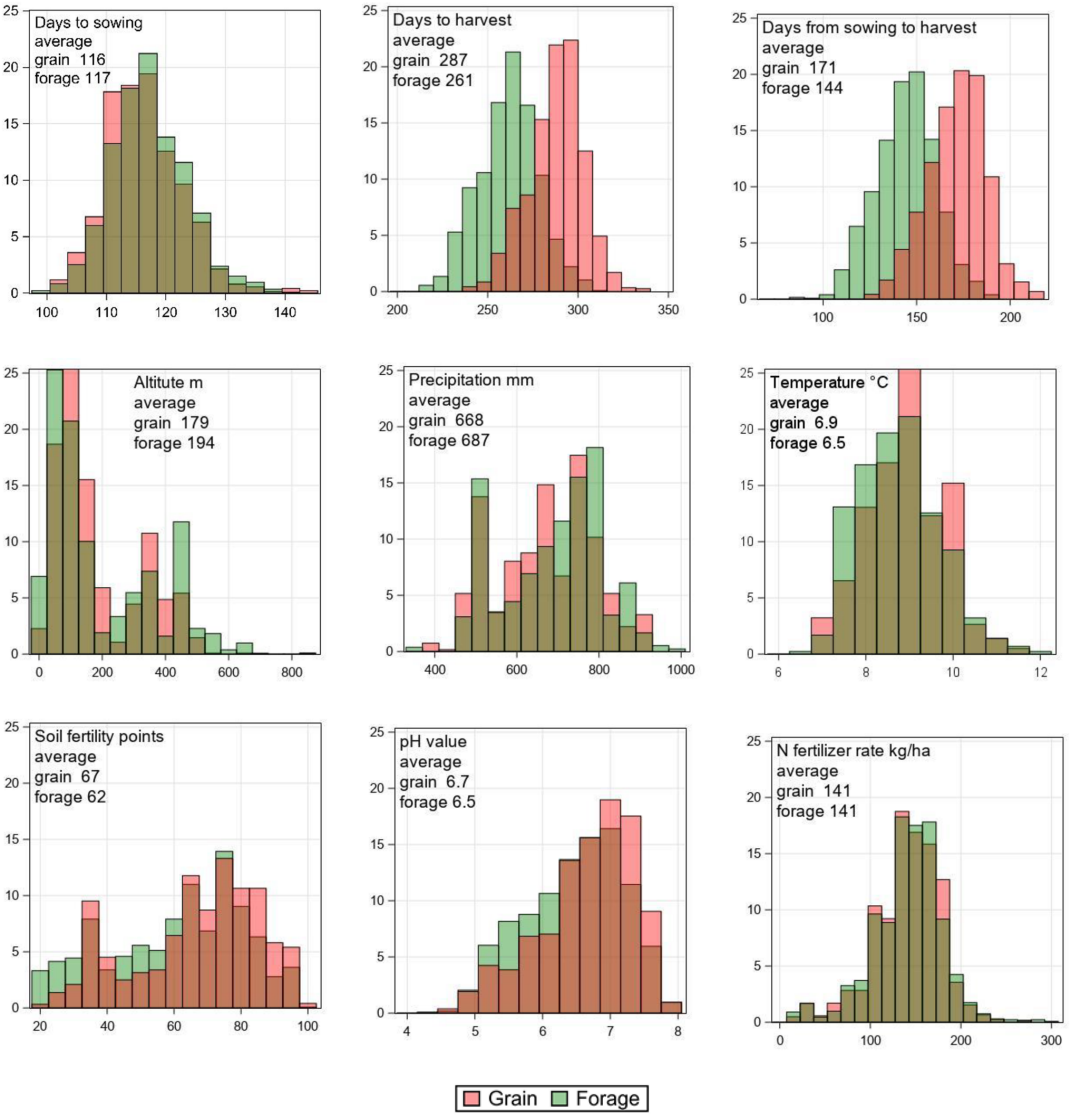
Frequency distribution of trial conditions for grain and forage maize medium maturity group based on trial observations. Days to sowing and harvest are counted from 1st of January. Temperature and precipitation are based on long-term annual averages for trial sites

Maize variety trials were conducted in the crop’s typical growing regions according to the climatic requirements of individual maturity groups across Germany. The geographical distribution of trial sites for grain and forage maize and their maturity groups across Germany is shown in Fig. 2. Generally, forage maize trial sites are more widespread across Germany than grain maize sites and the concentration of sites is moving southwards from early to late maturity groups. Trial site conditions for grain and forage maize cover a wide range as the frequency distributions of important parameters for the medium maturity group show (Fig. 3). Days from 1st of January to sowing was on average 116 and 117 days for grain and silage maize, respectively, while harvest of forage maize was on average 26 days earlier than for grain maize. Also, the number of days between sowing and harvest was 27 days earlier for forage maize than for grain maize. The average altitude of grain maize sites was 179 m and for forage maize 194 m above sea level, while the average long-term temperature, soil fertility points (In German: Ackerzahl) and pH value of the soil were lower for forage than for grain maize with 6.5 and 6.9 °C, 62 and 67 points, and 6.5 and 6.7, respectively. Average long-term precipitation for forage maize was slightly higher (687 mm) than for grain maize (668 mm) and N fertilizer rate was about the same with a mean of 141 kg ha^−1^. Maize VCU trials were integrated into typical crop rotation systems for maize crops with 57%, 14%, 23% and 6% share of cereals, maize, foliage (including tuber) crops and legumes grown as pre-crops, respectively (Data not shown). The frequency distributions of shown parameters indicate that VCU trials represent various agroclimatic conditions in Germany.

### Statistical analysis

#### Model for overall trend

For a given observation (average over replications), we used a model with factors genotype, sites, trial series and year. The overall model (Laidig et al. 2024) is given by

1 $$y_{ijkl} = \mu + L_{j} + Y_{k} + \left( {LYT} \right)_{jkl} + \left( {GY} \right)_{ik} + \left( {GLYT} \right)_{ijkl} ,$$

where *y*_*ijkl*_ is the mean yield of the *i*th genotype in the *j*th location and *k*th year within the *l*th trial series, *μ* is the overall mean, *L*_*j*_ is the main effect of the *j*th site, *Y*_*k*_ is the main effect of the *k*th year, *T* indicates the trial series (S1, S2, S3) and (*LYT*)_*jkl*_ is the effect of the *l*th trial series within the *jk*th location × year combination, (*GY*)_*ik*_ is the *ik*th genotype × year interaction effect and $\varepsilon_{ijkl}$ is a residual comprising the genotype × location × year interaction $\left( {GLY} \right)_{ijk}$, the genotype × location × year × trial series interaction $\left( {GLYT} \right)_{ijkl}$, and the error of a mean arising from sampling the replications. We confounded $\left( {GLY} \right)_{ijk}$ and $\left( {GLYT} \right)_{ijkl}$ with the residual error, because they were only based on the few reference varieties and were of about the same magnitude as the residual without these interactions (Hartung et al. 2023). The year effect $Y_{k}$ in the overall model represents the influence of environment and additionally the genotypes grown in this year, therefore we have not included a main effect for genotypes *G*_*i*_ and an interaction effect of genotype × site (*GY*)_*ik*_ in Eq. (1) because we consider *G*_*i*_ grown in the *k*th year to be hierarchically confounded with the year effect *Y*_*k*_ and the genotype × site effect (*GY*)_*ik*_ to be confounded with the residual effect. All effects are random. Further, we assume that *Y*_*k*_ is subject to a time trend representing a confounded genetic and non-genetic component, then Eq. (1) expands to

2 $$y_{ijkl} = \mu + \alpha_{1} t_{k } + \alpha_{2} t_{k}^{2} + L_{j} + Y_{k} + \left( {LYT} \right)_{jkl} + \left( {GY} \right)_{ik} + \left( {GLYT} \right)_{ijkl} ,$$

where $\alpha_{1}$ and $\alpha_{2}$ are fixed linear and quadratic regression coefficients for the overall trend, $t_{k}$ is the continuous covariate for the calendar year. The expected value under this model is given by

3 $$E\left( {y_{ijkl} } \right) = \mu + \alpha_{1} t_{k } + \alpha_{2} t_{k}^{2} .$$

Lodging plants were assessed as the ratio of the number of lodging plants and the total number of plants per two center rows. The trait was reported as percentage of lodging plants $y_{ijkl}$. As this measure cannot be assumed to be normally distributed, we used a pseudo-binomial model from the family of Generalized Linear Mixed Models (GLMM) allowing for over-dispersion (Piepho et al. 2024) to estimate adjusted year means and breeding progress. The logit link function was applied to the ratio $y_{ijkl} /100$ and for the error term a binomial distribution was assumed. The model effects are the same as given in Eqs. (1) and (2). We back-transformed the estimates provided by the GLMM with the inverse link function to get to the original scale, i.e., the percentage of lodging plants.

#### Estimation of breeding progress

The breeding progress achieved between $t_{k} =$ 1987 and 2023 was estimated based on the overall trend given by Eq. (3). The change between 2023 and 1987 was calculated as the difference of the predicted values for the years 2023 and 1987 by

4 $$\begin{gathered} Diff = E\left( {y_{ijkl} \mid t_{k} = 2023} \right) - E\left( {y_{ijkl} \mid t_{k} = 1987} \right) \hfill \\ = \alpha_{1} \left( {2023 - 1987} \right) + \alpha_{2} \left( {2023^{2} - 1987^{2} } \right){\text{using}}\;{\text{Eq}}.\;\left( {3} \right). \hfill \\ \end{gathered}$$

In the case that the quadratic regression term was not significant at α =  0.05, we used only a linear regression function. Also, for traits with *t*_*k*_ from 1999 to 2023, we used a linear regression function. Equations (2–4) have to be adjusted analogously.

#### Estimation of trend for trial site parameters

Observations related to trial sites like N fertilizer rate or days to harvest were modeled by

5 $$y_{jkl} = \mu + L_{j} + Y_{k} + \left( {LY} \right)_{jk} + \left( {LYT} \right)_{jkl} ,$$

where *y*_*jkl*_ is the observation of the *j*th site and the *k*th year within the *l*th trial series, *μ* is the overall mean, *L*_*j*_ is the main effect of the *j*th site, *Y*_*k*_ is the main effect of the *k*th year, *T* indicates the trial series and (*LYT*)_*jkl*_ the residual error. All effects are considered as random. Analogously to the overall model for estimating breeding progress, we also assume that the year effect *Y*_*k*_ is subject to a quadratic time trend with the covariable calendar year *t*_*k *_*,* then we can derive the change between 2023 and 1987 by using regression Eq. (4) analogously. In case that the quadratic regression term was not significant at α = 0.05, we assumed that the trend function is linear.

#### Assessing harvest index HI for grain maize

HI was estimated by merging data sets for grain and forage maize, because in routine VCU trials above ground dry matter yield is not assessed in grain maize. Only varieties which were grown together in both data sets at the same location and within the same year were used. Then for each variety HI was derived as the percent ratio of grain and dry matter yield relative to dry matter yield within location and year. However, the merged data set for HI has considerably less observations compared to the whole data set for grain and dry matter yield as shown in Table 1. Overall breeding progress was estimated as for the other traits by using the model given by Eq. (3). In years 1987 and 2023 only data from the first and second testing year with zero or only a few observations were available, therefore we dropped both years to obtain more stable regression estimates. However, to make differences in time trends comparable with other traits, we estimated the trend between 2023 and 1987 by Eq. (4).

#### Prediction of soil mineralized N (Nmin)

Soil mineralized N was assessed up to 60 cm soil depth at each trial site in spring before the start of vegetation. However, those data were only available for the years 2019–2023. We utilized the available data across grain, forage maize and maturity groups with *n* = 494 Nmin measurements to predict Nmin values for trials where no data were available. As possible predictor variables we used the categorial covariates grain maize, forage maize, maturity group, type of pre-crop, and as continuous covariates altitude, long-term average precipitation and temperature, soil fertility point, pH value and N fertilization rate. For the selection of covariates in the prediction model, we chose a step-wise backward procedure (Heinze et al. 2018). As selection criterion, the coefficient of determination *R*^2^ was applied (Piepho 2019). We stopped the selection of model terms until it reached *R*^2^ = 25.9% and could not be improved further ending up with the prediction model given by

5 $$y_{ijkl} = \mu + \left( C \right)_{i} +\alpha_{i} a_{ijkl} +\beta_{i} b_{ijkl} +\gamma_{i} g_{ijkl} + \delta_{i} d_{ijkl} + \eta_{i} n_{ijkl} + L_{j} + Y_{k} + \left( {LY} \right)_{jk} + \left( {CLYT} \right)_{ijkl}$$

where $y_{ijkl}$ represents the Nmin value measured at the *l*th trial series (S1, S2, S3) within the *k*th year, *j*th site and within three maturity groups of each grain and forage maize (*i* = 1, 2, …, 6). $\mu$ is the overall mean, $\left( C \right)_{i}$ is a fixed categorical effect for maturity group within grain and forage maize with six levels followed by five fixed regression coefficient and covariates for altitude of trial site $\alpha_{i}a_{ijkl}$, average long-term precipitation $\beta_{i} b_{ijkl}$, pH value $\gamma_{i} g_{ijkl}$ and soil fertility point $\delta_{i} d_{ijkl}$, respectively. The term $\eta_{i}$ is the coefficients for individual regression lines corresponding to the categorical effects $\left( C \right)_{i}$ for N fertilization rate $n_{ijkl}$, *L*_*j*_ is the main effect of the *j*th site, *Y*_*k*_ is the main effect of the *k*th year, *(YL)*_*jk*_ the interaction term year × site and $\left( {CLYT} \right)_{ijkl}$ is the residual error. We assumed that the effects *L*_*j*_, *Y*_*k*_, (*YL*)_*jk*_ and (C*LYT*)_*ijkl*_ are random and independent with constant variance, while all other effects are considered as fixed. The best linear unbiased predictor (BLUP) for Nmin of Eq. (5) was used to predict Nmin for all trials between 1987 and 2023 (Laidig et al. 2024).

## Results

**Fig. 4:**
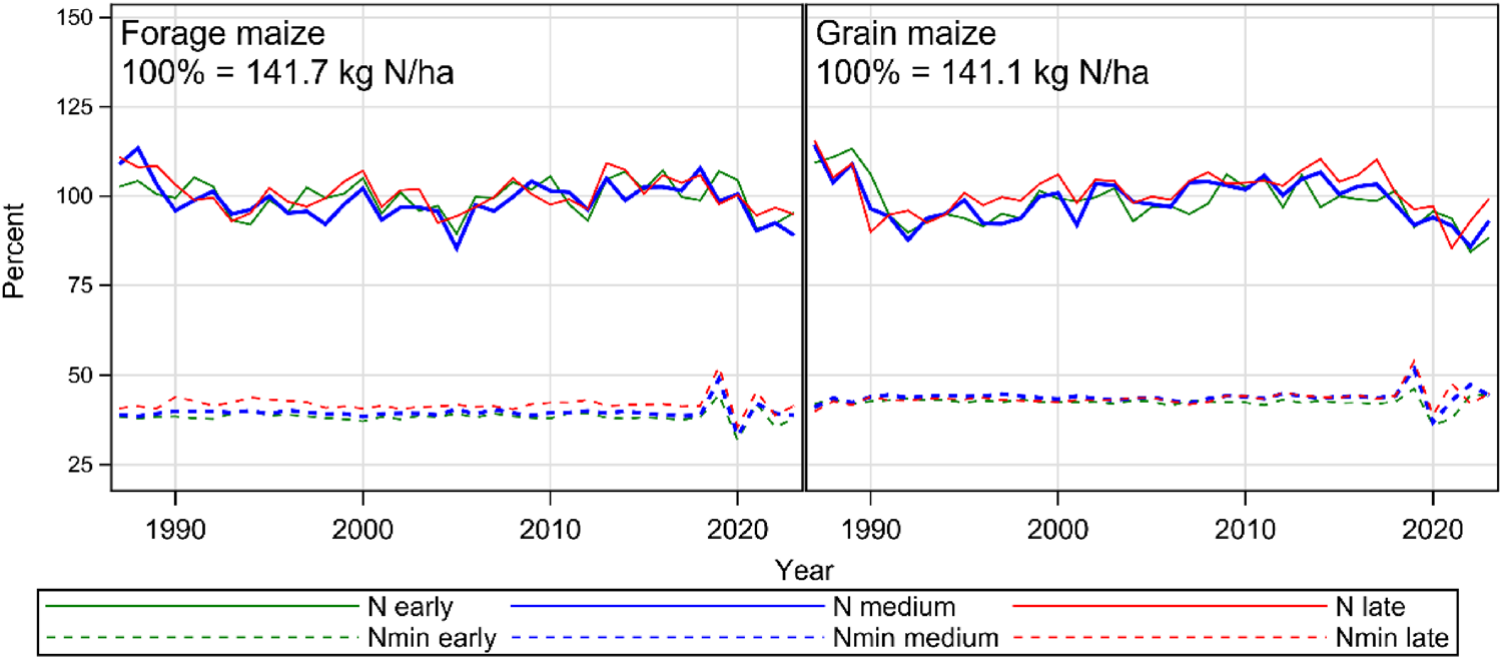
Trends for N fertilizer rate and soil mineralized N (Nmin) for early, medium and late maturity group based on adjusted year means. Adjusted year means correspond to least square means for years by considering the year effect in Eq. (1) as fixed

**Fig. 5:**
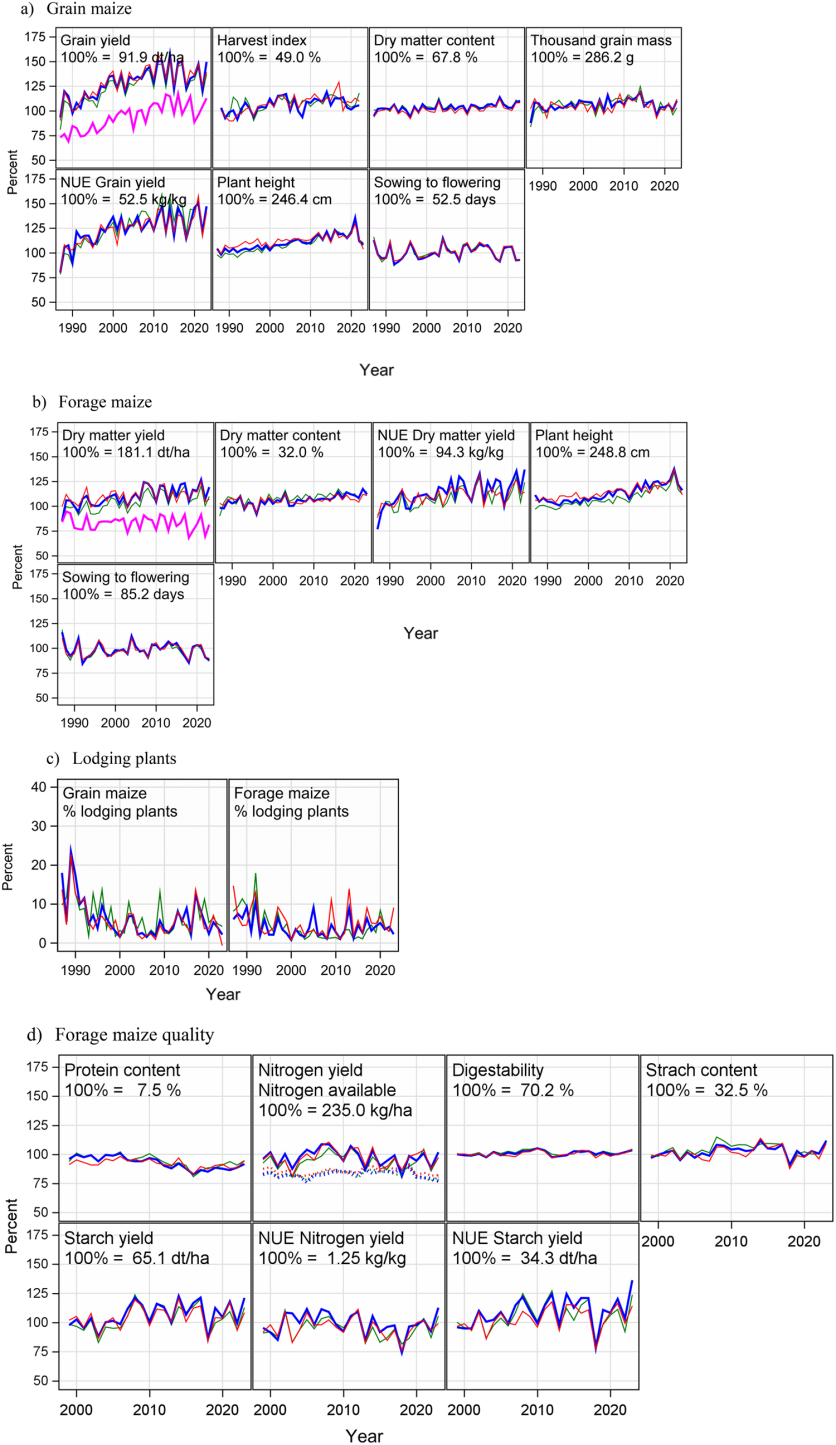
Trends of adjusted year means for **a** grain and **b** forage maize, and **c** percent of lodging plants at harvest as percent of total plot plants 1987–2023, and **d** quality traits of forage maize 1999–2023. Green lines correspond to early, blue to medium, red to late maturity group, dashed lines to available N fertilizer and pink to on-farm yields. Trends are shown relative to overall trend level 1987 (100%) for (**a**) and (**b**) and 1999 (100%) for **d** using (Eq. (4)). In Fig. 4c absolute percentage values of lodging plants are shown using the GLMM option in Eq. (1). Adjusted year means correspond to least square means for years by considering the year effect in Eq. (1) as fixed

Trends for adjusted year means for N fertilizer rates are depicted in Fig. 4, and trends for evaluated traits for grain and forage maize for all maturity groups are depicted in Fig. 5. In Table 2 the trend levels and breeding progress are shown only for medium maturity groups. We restricted the results in Table 2 to the medium group, because this was the group with the largest number of varieties (Table 1) and we further assume that the varieties of this group have the largest on-farm share and spread of growing area. Further, Fig. 5 indicates that the time trends of the early and late maturity group show very similar trend patterns with slightly different trait levels. Results for all maturity groups are shown in Supplemental Material Table S2.

**Table 2:** Levels of overall trends for traits of medium maturity group for grain maize 1987–2023, forage maize 1987–2023 and 1999–2023 and differences between levels 2023–1987 and 2023–1999 expressed in absolute (Diff) and relative (%) values based on levels 1987 and 1999 (Eq. (4))

Grain maize medium maturity 1987–2023							
Trait	Unit	1987	2023	Diff	%		Sign
Grain yield	dt ha^−1^	91.9	125.3	33.4	36.3	q	^***^
Grain yield on-farm	dt ha^−1^	64.2	97.0	32.8	51.0	q	^***^
Yield gap	%	30.1	22.6	− 7.5	−		
Harvest index	%	46.2	51.9	5.8	12.5	q	^**^
Dry matter content of grain	%	67.8	72.6	4.8	7.0		^***^
Thousand grain mass	g	286.2	298.3	12.2	4.3	q	ns
Plant height	cm	246.4	297.5	51.1	20.7		^***^
Lodging plants at harvest	%	10.1	4.9	− 5.2	− 51.3	q	^**^
N fertilizer rate	kg ha^−1^	141.1	137.9	− 3.2	− 2.3		ns
Soil minearlized N Nmin	kg ha^−1^	61.0	62.4	1.4	2.2		ns
NUE of Grain yield	kg kg^−1^	48.8	65.9	17.1	35.0		^***^

Forage maize medium maturity 1987–2023							
Trait	Unit	1987	2023	Diff	%		Sign
Dry matter yield	dt ha^−1^	181.1	217.2	36.1	19.9	q	^***^
Dry matter yield on-farm	dt ha^−1^	153.7	147.5	− 6.1	− 4.0		ns
Yield gap	%	15.1	32.1	16.9	–		
Dry matter content of whole plant	%	32.0	35.9	3.9	12.1		^***^
Plant height	cm	248.8	311.6	62.9	25.3		^***^
Lodging plants at harvest	%	3.8	4.0	0.2	6.2	q	ns
N fertilizer rate	kg ha^−1^	141.7	137.4	− 4.3	− 3.0		ns
Soil minearlized N Nmin	kg ha^−1^	55.7	56.2	0.5	0.9		ns
NUE of dry matter yield	kg kg^−1^	94.3	119.9	25.6	27.2		^***^

Forage maize medium maturity 1999–2023							
Trait	Unit	1999	2023	Diff	%		Sign
N yield in dry matter	kg ha^−1^	235.0	223.9	− 11.1	− 4.7		ns
N fertilizer rate	kg ha^−1^	140.2	137.4	− 2.9	− 2.0		ns
Soil minearlized N Nmin	kg ha^−1^	55.9	56.2	0.3	0.6		ns
Crude protein content	%	7.5	6.4	− 1.1	− 14.8		^***^
Digestibility	%	70.2	71.5	1.3	1.9		ns
Starch content	%	32.5	34.2	1.7	5.2		ns
Starch yield	dt ha^−1^	65.1	74.2	9.1	14.0		^*^
NUE of N yield	kg kg^−1^	1.3	1.2	0.0	− 2.0		ns
NUE of Starch yield	kg kg^−1^	34.3	40.6	6.3	18.3		^*^

q Quadratic regression function

Sign significance level; ^*^Significant at 5% level; ^**^Significant at 1% level; ^***^Significant at 0.1% level

### Time trends for N fertilization rate and predicted Nmin values

Trend lines for grain and forage maize in Fig. 4 show a similar year-to-year pattern indicating that both received about the same amount of N. Figure 4 further shows a slightly decreasing trend for forage maize and a stronger decreasing trend for grain maize. Trend levels in 1987 were 141.1 and 141.7, and in 2023 137.9 and 137.4 kg ha^−1^ for grain and forage maize, respectively, however this decay was not significant (Table 2). Moreover, the use of organic fertilizers such as liquid manure and digestives from biogas production increased in variety trials over time. For grain maize trials the share rose from 1.8 to 16.1% and for forage maize trials from 9.4 to 33.1% during 1987–2023 for the medium maturity group, respectively (Table S2). Predicted Nmin did not change significantly with levels 61.0 and 55.7 kg N ha^−1^ in 1987 and 62.4 and 56.2 kg N ha^−1^ in 2023 for grain and forage maize, respectively.

### Time trends for grain maize 1987–2023

Grain yield, the most important trait for grain maize increased significantly by 33.4 dt ha^−1^ (36.3% relative to 1987). The trend in Fig. 5a however showed a quadratic shape with an increase from 91.9 dt ha^−1^ then slowing down and nearly leveling off in 2023 at about 125.3 dt ha^−1^. The on-farm grain yields followed a similar pattern with nearly the same absolute increase of 32.8 dt ha^−1^ (51.0% relative to 1987), but at a considerably lower level of nearly 30 dt ha^−1^ in 2023 (Fig. 5a, pink line). The corresponding yield gap between VCU trials and on-farm was slightly reduced from 30.1% (1987) to 22.6% (2023, see Table 2). HI increased by 5.8% between 1987 and 2023 corresponding to a 12.5% increase relative to 1987. We observed larger year-to-year differences between maturity groups as compared to other traits which could possibly be attributed to fewer observations available per year. Dry matter content  of grain at harvest increased linearly by 4.8% (in absolute terms). Plant height increased strongly from 246.4 to 297.5 cm from 1987 to 2023 corresponding to an increase of 20.7%. Thousand grain mass increased until about 2015 and then dropped resulting in a non-significant change showing nearly a parallel pattern to grain yield. A strong increase of 35% was estimated for NUE of grain yield which was nearly as high as the increase in grain yield. The trend for percentage of lodging plants decreased by − 5.2% since 1987 in absolute terms. Figure 5c shows a decrease of lodging plants until 2010 and then large year-to year effects at low levels between 0 and 15% (Fig. 5c).

### Time trends for forage maize 1987–2023

Dry matter yield of forage maize increased less strongly than grain yield from 181.1 to 217.2 dt ha^−1^ by a nonlinear trend corresponding to a 19.9% increase relative to 1987. Noticeably, on-farm dry matter yields showed a non-significant decreasing linear trend of − 4.0% (153.7–147.5 dt ha^−1^) compared to 1987, which contrasts with the observed strong increase of on-farm grain yield. However, on-farm dry matter yields showed a similar year-to-year variation as in the VCU trials (pink lines in Fig. 5a and b). The diverging trend of VCU and on-farm forage maize yields resulted in a widened yield gap from 15.1% in 1987 to 32.1% in 2023. Dry matter content in the VCU trials increased significantly and linearly by 3.9% (in absolute terms). Plant height of forage maize (+ 62.9 cm or + 25.3%) increased stronger than for grain maize (+ 51.1 cm or + 20.7%). In contrast to grain maize, the trend of lodging plants of forage maize did not change significantly. As in grain maize, after a decrease until 2000, the percentage of lodging plants in forage maize ranged between 0 and 15% after 2000 showing larger year-to-year effects after 2010. NUE of dry matter yield in forage maize increased from 94.3 kg to 119.9 kg per kg of available N, which is a stronger absolute increase (25.6 kg kg^−1^) over time than for NUE of grain yield in grain maize (17.1 kg kg^−1^).

### Time trends for N yield and quality forage maize 1999–2023

Quality traits of forage maize to use as silage for feeding animals were assessed since 1999. We derived the accumulated N in the dry matter of the whole harvested plant by converting the protein content by the factor 1/6.25 and then multiplied this ratio with the dry matter yield as given in Table 2. Even though dry matter yield increased considerably by 36.1% from 1987 to 2023, the N yield was reduced by − 4.7% relative to 1999, however, the change was not significant. The protein content showed a clear negative trend (Table 2, Fig. 5d) with a level of 7.5% in 1999 and 6.4% in 2023 equivalent to a drop of − 14.8% relative to 1999. When comparing the sum of N applied by mineral and organic fertilizer plus the soil mineralized N (196.1 in 1999 and 193.6 kg ha^−1^ in 2023) (Table 2), more N was accumulated in harvested plants (235.0 in 1999 and 223.9 kg ha^−1^ in 2023) than available from fertilizer and Nmin. Furthermore, starch yield increased by 9.1 dt ha^−1^ or 14.0% while starch content did not change significantly but showed a positive trend of 1.7% (in absolute terms). Digestibility, which is an indicator for the quality of silage for feeding animals, did not improve significantly but increased by 1.3% or 1.9% relative to 1999. Compared to NUE for N yield, which decreased non-significantly by − 2.0% relative to 1999, NUE of starch yield increased considerably by 18.3% between 1999 and 2023 relative to 1999.

## Discussion

The discussion focuses on the results of the medium maturity groups. When results from the early and late maturity groups are discussed, this is explicitly mentioned.

### Breeding progress in yield and yield related traits of grain vs forage maize in VCU trials

Table 2 shows increasing grain yields for the medium maturity varieties by 36.3% (33.4 dt ha^−1^) within the last 36 years corresponding to an average annual increase of 0.93 dt ha^−1^ yr^−1^. This average increase is similar to the one in the early and late maturity group where it was 0.98 dt ha^−1^ yr^−1^ each (Table S2). In an earlier study by Laidig et al. (2014), the gain in grain yield from 1983–2012 for the medium maturity group was reported to be considerably higher, reaching 1.56 dt ha^−1^ yr^−1^. This smaller gain in our study can be explained by a leveling off or even drop of yields from about 2010 onwards as shown in Fig. 5a. Also, Mueller et al. (2019), DeBruin et al. (2017) reported from vintage trials in the US Corn Belt higher gains of 1.2 dt ha^−1^ yr^−1^ (1946–2015) and of 1.4 dt ha^−1^ yr^−1^ (1934–2013), respectively. However, Haegele et al. (2013) found lower gains of 0.79 dt ha^−1^ yr^−1^ at low N fertilizer level and 0.86 dt ha^−1^ yr^−1^ for high N levels during 1960 and 2010. In the observed period from 1987 to 2023 trends of decreasing average precipitation during April to October, more sunshine hours, increasing temperatures and an increasing carbon dioxide concentration have been observed in Germany. If and to which extent these factors may have contributed to the results of this study is not clear (Figs. 1b and 5). N fertilizer rate decreased slightly by − 3.2 kg ha^−1^ in this period. Even though the decline was not significant, it still contrasts with rising yields and the corresponding presumable increase in nitrogen demand. Even more significant is the increasing share of organic fertilizer in grain and forage maize trial series (Table S2). Results from field and laboratory incubation experiments provide evidence that N availability to plants is generally lower in organic compared to mineral fertilizer. Different studies show that first-year crop availability of slurry N comes mainly from its ammonium fraction because net mineralization of organic N is often negligible in the short term leading to a lower N uptake efficiency of organic N fertilizer compared to mineral N fertilizer (Cavalli et al. 2014; Pantelopoulos et al. 2017; Bachmann et al. 2011). Despite the stagnation in grain and dry matter yield from 2010 on, considerable gains were reached over the entire investigation period. Average plant height increased in grain and forage maize by 51.1 and 62.9 cm (1987–2023), respectively, which can be an indirect effect of selection for higher yields. A possible explanation for the higher increase in plant height in forage maize is that more or different forage type varieties were included in the forage trial series. Breeding for higher resistance toward lodging was successful, implying less risk of respective yield losses, which is in line with Smith et al. (2014) who reported lessening stalk lodging in US maize. Besides taller plants, other yield- relevant characteristics like larger leaf area and steeper leaf angle increased radiation efficiency have contributed to breeding progress according to Taube et al. (2020) and Messina et al. (2022).

In contrast to grain yield, dry matter yield for the medium maturity group increased by 19.9% (36.1 dt ha^−1^) for forage maize corresponding to 1.00 dt ha^−1^ yr^−1^ (recalculated by 36.1 dt ha^−1^/36 years). The progress in the early maturity group was 1.00 dt ha^−1^ yr^−1^_,_ but in the late group only 0.46 dt ha^−1^ yr^−1^. The lower gain in the late maturity group could be attributed to the higher yield levels of the late group in 1987 compared to the early and medium maturity groups, while yield level in 2023 was between the early and the medium group (Table S2). For dry matter yield, we observed a reduced rate of increase from 2010 on, however, lower than for grain maize. Our results for the early and the medium group are in line with the reported increase in dry matter yield by Taube et al. (2020) of 1.1 dt ha^−1^ yr^−1^ from a vintage study of 10 maize genotypes with year of release from 1971 to 2012 in North-West Germany. In contrast to the results of Taube et al. (2020) and our study, Mackay et al. (2011) and Schils et al. (2020) reported a higher increase for national VCU trials comprising all maturity groups which was around twice as high with 2.47 dt ha^−1^ yr^−1^ and of 2.12 dt ha^−1^ yr^−1^ for the UK (1982–2007) and the Netherlands (1990–2016), respectively. One possible reason for the higher gains in the UK and the Dutch VCU trials may also be attributed to the fact that years from 2008 and 2017 were not included, while in Germany dry matter yield stagnated from 2010 onwards (Fig. 5a). Further, Germany experienced a very dry decade during the 2010s (DWD, 2025b), while in the Netherlands, where annual rainfall is generally slightly higher, it was not that dry during the same period (KNMI 2025).

The question arises, why the yield gain of forage maize (19.9%) is considerably lower compared to grain maize (36.3%) in German VCU trials? One possible explanation can be ascribed to the progress of HI of 6.5%, 5.8% and 10.8% for early, medium and late maturity groups corresponding to an annual increase of 0.14% yr^−1^, 0.16% yr^−1^ and 0.30% yr^−1^, respectively, indicating that breeding was successful for an increased partitioning and translocation from stem and leaves to grain. Similar gains of HI as for the early and medium maturity group in our study were reported by Ruiz et al. (2023) for US Corn Belt genotypes of 0.17% yr^−1^ for early and 0.13% yr^−1^ for later maturing genotypes between 1983 and 2020. They ascribe this gain fully to genetic improvement. Further, Ruiz et al. (2023) combined 16 different literature data sets from different countries based on a large range of environments and genotypes and found an average increase in HI of 0.25% yr^−1^ varying within a large range of 0.08% yr^−1^ and 0.47 yr^−1^ since 1964. Reported increases in HI indicate that progress was achieved, against Fischer and Edmeades (2010) who considered the prospects of further increase of HI as low. HI is not a target for practical maize breeders nor is it a trait in the framework for testing VCU in registration trials. However, HI can be considered as result of indirect selection for higher yield and it is of general interest to understand the drivers of breeding progress in maize (Ruiz et al. 2023; Smith et al. 2014).

In addition, the lower gain in forage maize compared to grain maize is likely because the geographical distribution of forage trial sites has shifted more into growing areas of Germany with possibly less favorable growth conditions for maize (Fig. 2). Compared to grain maize, forage maize trial sites had a lower average daily temperature and soil fertility points were lower. However, management and growth conditions were like grain maize with respect to N rate, date of sowing, average precipitation, plant density, and share of pre-crops (Fig. 3, Table S1).

### Yield gaps

The strong breeding progress observed in trials is not always fully translated into increasing on-farm yields (Laidig et al. 2014; Mackay et al. 2011; Schils et al. 2020). Yield gaps for grain and forage maize between VCU trials and national on-farm yields are depicted in Fig. 5 and quantified in Table 2. The yield gap for grain maize reduced by − 7.5% from 30.1% (1987) to 22.6% (2023). While VCU dry matter yields increased, on-farm dry matter yield for forage maize, however, decreased by − 6.1 dt ha^−1^, corresponding to − 0.17 dt ha^−1^ yr^−1^. Contrary to the on-farm yield stagnation of forage maize in Germany, on-farm yields in the Netherlands showed significant positive trends for forage maize with 1.95 dt ha^−1^ yr^−1^ (Schils et al. 2020). This stagnation resulted in an increased gap in Germany from 15.1% (1987) to 32.1% (2023). While it is well known that on-farm yields do not reach trial results on average (e.g., Fischer and Edmeades 2010; Mackay et al. 2011; Schils et al. 2020; Smith et al. 2014), the question arises, how strongly maize yield gaps can be narrowed? Fischer et al. (2014) argue that a yield gap of 30% based on farm level, equivalent to about 25% on trial level (as applied in our study), might be economically attainable, whereas Lobell et al. (2009) set the benchmark to 20% in developed agriculture. Smith et al. (2014) stated that the average national yields gap in the US tend to plateau at 15–25% of attainable yield. Considering 20% as a baseline, our study revealed potential for improvement of on-farm forage maize yields in Germany. Our results have shown that the gap in forage maize increased considerably compared to grain maize raising the question why this gap is much larger than 20%. The reasons are complex, and their detailed investigation is beyond the scope of this study. Generally, influencing factors are crop management, production sites, climatic change and agronomic policy goals, like fertilizer regulations. A major factor to consider is the increasing demand of forage maize for biogas production from 2005 on, which nearly doubled the growing area (Fig. 1a) and consequently extended it to environments with less favorable conditions, including sites with lower soil fertility, higher altitude and lower mean temperatures. Further, forage maize used for biogas production may be grown in crop rotations with higher frequency of maize-after-maize cropping, with negative impacts on pests and diseases as well as soil properties, which may additionally be impaired by heavy machinery.

Another hampering factor for higher input may be the strongly increasing costs for fertilizer and other means of production resulting in an economically viable lower input intensity in recent years. Further, the increasing substitution of mineral N by biogas digestate may also have had an influence on stagnating on-farm yields, due to the generally lower plant availability of organic compared to mineral N (Bachmann et al. 2011; Cavalli et al. 2014; Pantepoulos et al. 2017), because about 45% of total maize acreage is used for biogas production (FNR 2025). Another important factor may be the farmers’ choice of the best variety, which has many aspects. First, the introduction of new varieties in practice lags at least three years behind (Schils et al. 2020), second, the choice by farmers is influenced by many factors other than yielding ability (Macholdt and Honermeier 2016). Unfortunately, access to long-term farm survey data regarding the distribution of forage vs. grain maize including maturity groups, specific varieties and crop management are not available and a detailed analysis is beyond the scope of this study.

### Nitrogen use efficiency for grain and forage yield (1987–2023)

We derived NUE as a percentage of grain and forage yield relative to the available N fertilizer (N fertilizer + Nmin in soil). Our results are assessed over a wide range of genotypes, environments and N rates with a large variability of individual NUE values. NUE of grain yield was considerably higher reaching 48.8 kg kg^−1^ (1987) and 65.9 kg kg^−1^ (2023) compared to Mueller et al. (2019) of 17 kg kg^−1^ (1976) and 30 kg kg^−1^ (2015). Similarly, Gheith et al. (2022) reported NUE values in the range of 12.5–22.3 kg kg^−1^ from a study based on one hybrid, four N levels and three timings of N-application in two years. The lower NUE of Müller et al. (2019) may be attributed to their use of the “difference method” (Yan et al. 2019), which calculates NUE as the yield from N-fertilization minus yield from zero-N divided by the applied N rate. This approach may underestimate NUE by excluding nitrogen from mineralization and residue turnover, especially in systems with long-term rotations. In contrast, our method incorporates all available N sources over a crop’s growing period, reflecting more realistic uptake patterns. Yan et al. (2019) found that only 41% of maize N uptake came from current-year fertilizer, with the remainder from soil and residue turnover. This aligns with findings that over 50% of applied N is either immobilized in soil or lost, yet not entirely unavailable, because some becomes accessible to subsequent crops (Cassman et al. 2002; Ciampitti et al. 2012; Lassaletta et al. 2014). Our approach accounts for N mineralization from previous crop residues and soil organic matter, which also benefits the zero-fertilizer treatments, therefore the NUE from the difference method provides lower NUE values than the method we used.

### Quality and N accumulation in dry matter of forage maize (1999–2023)

Regarding quality parameters of forage maize, significant positive trends were found for starch yield (+ 14,0% relative to 1999) and NUE of starch yield (+ 18,3% relative to 1999), but in the medium maturity group only. Protein content decreased by − 14.8% relative to the trend level in 1999 (Table 2). This negative trend was also found to be significant in the other two maturity groups (Table S2). Trends of all other quality parameters showed no significant change. For starch content and crude protein content our results are in line with Taube et al. (2020) who found no significant change for starch content and a negative trend for protein content. Although trends for digestibility and starch content were not found to be significant, both parameters show positive signs of the change, which is consistent in all three maturity groups (Table S2). Considering that in parallel total dry matter yields showed significant gains, an interpretation of the results of our study could be that the product of quality and yield, i.e., starch yield, also showed breeding progress. At least one could conclude that the two forage quality parameters starch content and digestibility (+ 5.2% for starch content and + 1.9% for digestibility relative to 1999), have not been negatively affected during the period of the study while dry matter yields showed significant gains. However, the trend of starch yield, supporting this hypothesis, was only found to be significant in the medium maturity group and not in the early and late maturity group. Protein content in plant dry matter decreased significantly from 7.5 (1999) to 6.4% (2023) in line with Mueller et al. (2019) reporting also a strong decrease from 8.4 (1958) to 6.7% (2015), and Taube et al. (2020) of similar magnitude.

In our study, the decline in protein content could apparently not be offset by the increasing dry matter yield to lead to an overall increase in nitrogen yield. Consequently, the NUE of N yield was also not changed significantly. Despite the result that no gain for N yield was found, the accumulated N in dry matter of 235.0 kg ha^−1^ (1999) and 223.9 kg ha^−1^ (2023) was higher than the available N of 196.1 kg ha^−1^ (1999) and 193.6 kg ha^−1^ (2023), corresponding to an additional N mobilization of 38.9 kg ha^−1^ (1999) and 30.3 kg ha^−1^ (2023). This difference indicates that a high level of total plant N uptake was achieved in the datasets analyzed and additional mineralized nitrogen from the soil was taken up by the plants implying a reduced N loss and consequently lower negative environmental impacts.

## Conclusions

Our analyses revealed a strong breeding progress in maize VCU trials in Germany from 1983 to 2023 of 36.3% for grain maize yield and 19.9% for forage maize dry matter yield relative to 1987, however, trends indicated a slowing down of the upward trend during the last 10–15 years. At the same time, volatility of annual yield levels increased strongly, especially for grain maize. Increased HI is considered as one possible factor contributing to the yield gain in grain maize. Despite taller plants, breeding decreased the incidence of lodging and hence reduced the risk of yield losses at harvest. Furthermore, the results of our study show that the described yield gains prevail despite constant N fertilizer rates over the 36-year period of the study. This resulted in a strong increase of NUE for grain and dry matter yield of 35.0% and 27.2%, relative to 1987, respectively. While the yield gap between VCU trials and on-farm yield significantly narrowed from 30.1 to 22.6% in grain maize, the yield gap in forage maize increased remarkably from 15.1 to 32.1%. As forage maize covers more than 75% of total maize acreage in Germany, it is of vital importance for practical maize production to prevent a widening or even achieve a reduction in this gap. Possible reasons for this increasing yield gap are the rapid extension of forage maize acreage from 2005 onwards, most likely expanding production to regions with less favorable growing conditions and possibly less favorable crop rotations. Further, increasing prices likely led to a decreasing production intensity, especially regarding N fertilizer, not aiming at highest yields but the optimal economic intensity. Moreover, an increase in the share of organic N sources, especially biogas digestates over mineral N may have hampered N availability to plants. Starch yield for forage maize gained 14.0% (1999–2023), however, no significant trend was found for starch content and digestibility. Nevertheless, these two trends of quality parameters showed positive signs, indicating that quality parameters have not been negatively affected while dry matter yields increased. N yield accumulated in dry matter decreased non-significantly, while N uptake exceeded the available N fertilizer by 38.9 kg ha^−1^ (1999) and 30.3 kg ha^−1^ (2023) indicating high total plant N uptake efficiency, and hence a reduced N loss lowering negative environmental impact. Our study has shown that breeding progress of grain maize was successfully transformed into farm yield, but a considerable gap remains between the yield potential of new varieties and on-farm yield especially for forage maize. Additional research is required to assess the specific reasons for the widening yield gaps in forage maize and to identify levers to prevent this widening effectively.

## Supplementary Information

Below is the link to the electronic supplementary material.

Supplementary file1 (PDF 118 KB)

Supplementary file2 (PDF 175 KB)

## Data Availability

Data were provided by the Federal Plant Variety Office for exclusive use in this study and are in general not publicly available. Reasonable requests may be addressed to the Federal Plant Variety Office, Hannover, Germany.
