# Supplementary material for: Breeding progress of grain and forage maize in long-term variety trials compared to on-farm yield development
Source: Theor Appl Genet. 2025 Nov 13;138(12):303. doi: 10.1007/s00122-025-05085-6 (PMC12612010; doi:10.1007/s00122-025-05085-6)
Supplement: Supplementary file 1 — Supplementary file1 (PDF 118 KB) [file 122_2025_5085_MOESM1_ESM.pdf]

Table S1 Levels of overall trends for a) trial and b) climatic conditions (average April – October) for years 1987 and 2023 and difference between levels 2023 and 1987 expressed in absolute (Diff) and relative (%) values based on level 1987 (%) (Eq. (4)).

a)

Traits		Grain maize						Forage maize					
	Maturity	1987	2023	Diff	%	sig		1987	2023	Diff	%	sig	
Days from first of January to day of sowing	early	118.1	116.2	–2.0	–1.7	ns		119.1	118.2	–1.0	–0.8	ns	
	medium	118.3	115.9	–2.5	–2.1	ns		118.6	116.2	–2.4	–2.0	ns	q
	late	118.1	116.2	–2.0	–1.7	ns		118.4	115.0	–3.4	–2.9	*	
Days from first of January to day of harvest	early	290.0	288.4	–1.6	–0.6	ns		268.5	258.9	–9.6	–3.6	ns	
	medium	289.7	285.9	–3.9	–1.3	ns		269.5	257.1	–12.4	–4.6	*	
	late	289.0	285.8	–3.2	–1.1	ns		266.0	253.1	–12.9	–4.9	*	
Days from sowing to harvest	early	171.6	172.5	0.9	0.5	ns		149.1	140.8	–8.4	–5.6	ns	
	medium	170.9	170.2	–0.7	–0.4	ns		150.9	140.7	–10.2	–6.7	ns	
	late	170.5	169.9	–0.5	–0.3	ns		147.4	138.6	–8.8	–6.0	ns	
Soil fertiliy points	early	59.7	63.4	3.7	6.2	***	q	54.8	58.7	4.0	7.2	***	q
	medium	60.6	66.4	5.9	9.7	***	q	56.8	61.4	4.7	8.2	***	q
	late	61.2	66.7	5.5	8.9	***	q	61.8	67.3	5.5	8.9	***	q

b)

Climate (average April – December)	1987	2023	Diff	%	sig	
Average yearly precipitation mm	457.4	426.8	–30.6	–6.7	ns	
Average yearly temperature °C	13.2	14.7	1.5	11.5	***	
Cumulative ýearly sunshine hours h	1247.1	1443.2	196.2	15.7	**	
CO2 concentration in atmosphere ppm	349.8	422.1	72.3	20.7	***	q

q Quadratic regression function
Sign significance level; * Significant at 5% level; ** Significant at 1% level; *** Significant at 0.1% level;
