# Supplementary material for: Breeding progress of grain and forage maize in long-term variety trials compared to on-farm yield development
Source: Theor Appl Genet. 2025 Nov 13;138(12):303. doi: 10.1007/s00122-025-05085-6 (PMC12612010; doi:10.1007/s00122-025-05085-6)
Supplement: Supplementary file 2 — Supplementary file2 (PDF 175 KB) [file 122_2025_5085_MOESM2_ESM.pdf]

Tab S2 Levels of overall trends of traits for a) grain maize 1987-2023 and b) forage maize 1987–2023 and c) forage maize 1999–2023 and difference between levels 2023 and 1987 and 1999 expressed in absolute (Diff) and relative (%) values based on levels of 1987 and 1999 (Eq. (4)).

a)

			Grain maize 1987-2023					
		Maturity	1987	2023	Diff	%		Sign
Grain yield	dt ha^{-1}	early	85.2	120.6	35.4	41.5	q	***
		medium	91.9	125.3	33.4	36.3	q	***
		late	89.7	124.9	35.3	39.3	q	***
		on-farm	64.2	97.0	32.8	51.0	q	***
Yield gap	%	early	24.6	19.6	–5.1			
		medium	30.1	22.6	–7.5			
		late	28.4	22.3	–6.0			
Harvest index		early	48.1	54.6	6.5	13.6	q	**
		medium	46.2	51.9	5.8	12.5	q	**
		late	47.2	58.0	10.8	22.9		***
Dry matter content	%	early	68.4	72.4	3.9	5.8		**
		medium	67.8	72.6	4.8	7.0		***
		late	66.6	71.0	4.4	6.6		**
Thousand grain mass		early	279.5	294.1	14.6	5.2	q	ns
		medium	286.2	298.3	12.2	4.3	q	ns
		late	297.0	304.2	7.2	2.4		ns
Plant height	cm	early	235.9	299.9	64.0	27.2		***
		medium	246.4	297.5	51.1	20.7		***
		late	260.2	292.7	32.4	12.5		***
Lodging plants at harvest		early	9.4	5.3	–4.1	–43.3	q	ns
		medium	10.1	4.9	–5.2	–51.3	q	**
		late	9.8	4.8	–5.0	–50.9	q	*
Days from sowing to flowering	d	early	81.2	82.5	1.3	1.6		ns
		medium	80.9	82.0	1.1	1.4		ns
		late	82.5	81.5	–1.0	–1.2		ns
N fertilizer rate kg	kg ha^{-1}	early	143.0	133.5	–9.5	–6.7		ns
		medium	141.1	137.9	–3.2	–2.3		ns
		late	143.1	142.9	–0.1	–0.1		ns
Organic N fertilizer, % total N	%	early	4.1	27.3	23.2			***
		medium	1.8	16.1	14.3			***
		late	2.7	16.1	13.4			***
Soil mineralized N Nmin	kg ha^{-1}	early	60.4	59.4	–1.0	–1.7		ns
		medium	61.0	62.4	1.4	2.2		ns
		late	59.4	62.9	3.6	6.0		ns
NUE of grain yield	kg kg^{-1}	early	44.3	68.1	23.8	53.7	q	***
		medium	48.8	65.9	17.1	35.0	q	***
		late	47.7	64.3	16.6	34.7	q	***

b)

		Forage maize 1987-2023					
		Maturity	1987	2023	Diff	%	Sign
Total dry matter yield	dt ha^{-1}	early	172.0	205.7	33.7	19.6	***
		medium	181.1	217.2	36.1	19.9	***
		late	192.5	209.2	16.7	8.7	*
		on-farm	153.7	147.5	–6.1	–4.0	ns
Yield gap	%	early	10.6	28.3	17.6		
		medium	15.1	32.1	16.9		
		late	20.2	29.5	9.3		
Dry matter content	%	early	32.7	36.7	3.9	12.1	***
		medium	32.0	35.9	3.9	12.1	***
		late	32.9	34.7	1.8	5.6	**
Plant height	cm	early	237.8	298.6	60.7	25.5	***
		medium	248.8	311.6	62.9	25.3	***
		late	260.4	308.1	47.7	18.3	***
Lodging plants at harvest		early	7.7	2.8	–4.9	–63.5	**
		medium	3.8	4.0	0.2	6.2	ns
		late	6.1	5.6	–0.5	–7.8	ns
Days from sowing to flowering	d	early	84.3	82.0	–2.4	–2.8	ns
		medium	85.2	82.9	–2.2	–2.6	ns
		late	84.9	82.9	–2.0	–2.3	ns
N fertilizer rate	kg ha^{-1}	early	141.4	140.9	–0.5	–0.4	ns
		medium	141.7	137.4	–4.3	–3.0	ns
		late	144.4	140.4	–4.0	–2.8	ns
Organic N fertilizer, % total N	%	early	9.4	42.3	32.9		***
		medium	9.4	33.1	23.7		***
		late	5.3	19.2	13.9		***
Soil mineralized N Nmin	kg ha^{-1}	early	54.5	54.0	–0.5	–0.9	ns
		medium	55.7	56.2	0.5	0.9	ns
		late	59.2	59.4	0.3	0.4	ns
NUE of dry matter yield	kg kg^{-1}	early	92.4	111.6	19.2	20.8	***
		medium	94.3	119.9	25.6	27.2	***
		late	97.5	111.8	14.3	14.7	**

c)

		Forage maize 1999-2023					
		Maturity	1999	2023	Diff	%	Sign
Crude protein content in DM	%	early	7.4	6.6	–0.8	–11.4	***
		medium	7.5	6.4	–1.1	–14.8	***
		late	7.1	6.6	–0.5	–6.6	*
N fertilizer rate	kg ha^{-1}	early	141.3	140.9	–0.4	-0.3	ns
		medium	140.2	137.4	–2.9	–2.0	ns
		late	143.1	140.4	–2.7	–1.9	ns
Soil mineralized N Nmin	kg ha^{-1}	early	54.3	54.0	-0.3	-0.6	ns
		medium	55.9	56.2	0.3	0.6	ns
		late	59.3	59.4	0.2	0.3	ns
N yield in DM	dt ha^{-1}	early	222.9	220.5	–2.4	–1.1	ns
		medium	235.0	223.9	–11.1	–4.7	ns
		late	230.6	226.3	–4.4	–1.9	ns
Digestibility	%	early	70.9	71.8	1.0	1.3	ns
		medium	70.2	71.5	1.3	1.9	ns
		late	70.1	71.0	0.9	1.2	ns
Starch content	%	early	33.4	34.4	1.0	2.9	ns
		medium	32.5	34.2	1.7	5.2	ns
		late	32.4	33.4	1.0	3.1	ns
Starch yield	dt ha^{-1}	early	64.1	70.9	6.8	10.6	ns
		medium	65.1	74.2	9.1	14.0	*
		late	66.9	69.0	2.1	3.2	ns
NUE of n yield in DM	kg kg^{-1}	early	1.20	1.18	-0.03	–2.32	ns
		medium	1.25	1.23	–0.03	–2.01	ns
		late	1.22	1.18	–0.04	–3.10	ns
NUE of starch yield	kg kg^{-1}	early	34.4	37.8	3.4	9.7	ns
		medium	34.3	40.6	6.3	18.3	*
		late	34.1	36.8	2.7	7.9	ns

q Quadratic regression function

Sign significance level; * Significant at 5% level; ** Significant at 1% level; *** Significant at 0.1% level;
